# Proteomics and phosphoproteomics reveal novel proteins involved in *Cipangopaludina chinensis* carcasses

**DOI:** 10.3389/fchem.2024.1416942

**Published:** 2024-08-29

**Authors:** Gongzhen Liu, Kangyu Zhong, Shanmin Gong, Xinru Li, Yanshen Li

**Affiliations:** ^1^ College of Agriculture and Forestry, Linyi University, Linyi, China; ^2^ Department of Marine Product Quality and Safety Inspection Key Laboratory, Yantai University, Yantai, China

**Keywords:** proteomics, Phosphoproteomics, *Cipangopaludina chinensis*, China, Kyoto Encyclopedia of Genes and Genomes

## Abstract

*Cipangopaludina chinensis* is a common freshwater mollusk that is widely distributed worldwide, especially in China. In our research, 1,382 proteins and 1,039 phosphorylated proteins were identified from *C. chinensis* carcasses, and 690 differentially expressed proteins (DEPs) were quantified. Gene Ontology (GO) and Kyoto Encyclopedia of Genes and Genomes (KEGG) analyses revealed that the DEPs are involved in cellular processes, single-organism processes, metabolic processes, developmental processes, localization, and biological regulation. The phosphorylated proteins were found to be related to the Rap1 signaling pathway, Ras signaling pathway, calcium signaling pathway, and longevity-regulating pathways. Moreover, we also identified important regulatory enzymes, such as guanylate cyclase, tyrosine protein kinase, receptor protein tyrosine kinase, and glyoxylate reductase/hydroxypyruvate reductase. Notably, we found guanylate cyclase to be present in multiple signaling pathways, including the Rap1 signaling pathway, calcium signaling pathway, Ras signaling pathway, insulin secretion, longevity regulating pathway, glutamatergic synapse, circadian entrainment, and gap junction. This enzyme may play a crucial role in regulating molecular mechanisms in *C. chinensis.* In summary, proteomic and phosphoproteomic analyses of *C. chinensis* carcasses displayed significant differences among different geographical isolates, which helps enhance our understanding of food nutrition, signaling pathways, and metabolic mechanisms in *C. chinensis*.

## 1 Introduction

*Cipangopaludina chinensis* (*C. chinensis*) is a common mollusk that inhabits freshwater environments and is widely distributed worldwide. It is commonly found in rice fields, lakes, swamps, rivers, ditches, and paddy fields, among other areas. Since it is particularly easy to find in paddy fields, it is known as the field snail (Chen, 2019; Luo et al., 2021; Zhou et al., 2021). Taxonomically, *C. chinensis* belongs to the phylum Mollusca, Gastropoda, Prosobranchia, Mesogastropoda, Architaenioglossa, Viviparidae, and Cipangopaludina. It is closely related to another species, *Cipangopaludina cathayensis* (*C*. *cathayensis*) (Table 1) (Pakkasmaa, 2001; Wang and Xie, 2004).The relationship between humans and snails is quite intimate: 1) *C. chinensis*, a freshwater snail that still exists in a wild or semi-wild state, boasts a white and sweet carcass, unique flavor, and rich content of protein and fat (Xiong et al., 2019; Luo, 2022). 2) It serves as essential natural food bait for livestock, poultry, fish, and aquatic products. 3) However, due to their large numbers, snails can cause blockages in the cooling water pipes of lakeside enterprises. 4) Additionally, coupled with the invasion of alien species like *Pomacea canaliculata*, the development of the native snail breeding industry has been sluggish, making it urgent to conduct related genetic and breeding studies. According to the Red List of Species of China, there are 40 threatened snail species, including 6 critically endangered, 22 frequently endangered, and 12 vulnerable species (Wang and Xie, 2004). 5) Furthermore, snails are valuable teaching material in zoology, essential for studies in ecology and taxonomy. 6) In traditional Chinese medicine, snail carcasses have numerous therapeutic benefits, including nourishing the kidneys, improving vision, quenching thirst, clearing heat, promoting diuresis, treating kidney deficiency fatigue, eye heat and redness, urination obstruction, otitis media, hemorrhoids, jaundice, and body odor (Zhou et al., 2022a; Zhou et al., 2022b; Wu et al., 2022; Zhao et al., 2022; Rees et al., 2023).

**TABLE 1:** Viviparidae snails in China (Wang et al., 2004).

Species	Year	Environment	Area	Degree
*Viviparus tricinctus*	Liu et al. (1994)	River snail	Endemic to China; Hunan and Guizhou	EN
*Viviparus chui*	Yen (1937)	River snail	Endemic to China; Heilongjiang and Jilin	EN
*Cipangopaludina Chinensis*	Grey (1834)	Pond snail	Major range in China; common in China except Xinjiang and Xizang	LC
*Cipangopaludina cathayensis*	Heude (1890)	Pond snail	Endemic to China	LC
*Cipangopaludina ampullacea*	Charpantier (1863)	Lake snail	Endemic to China; Sichuan and Yunan	VU
*Cipangopaludina fluminalis*	Heude (1890)	River snail	Endemic to China; N, SE, and SW	LC
*Cipangopaludina ampulliformis*	Souleyet (1852)	River snail and pond snail	Major range in China; Hainan and Fujian	VU
*Cipangopaludina aubryana*	Heude (1890)	Pond snail	Major range in China; SW and SE	VU
*Cipangopaludina hassi*	Prashed (1928)	Lake snail	Endemic to China; Yunnan and Sichuan	VU
*Cipangopaludina lattissima*	Dautzenberg and Fisher (1905)	Lake snail	Endemic to China; Yunnan	EN
*Cipangopaludina lecythis*	Benson (1836)	Pond snail	Endemic to China; Yunnan	CR
*Cipangopaludina lecythoides*	Benson (1842)	Lake snail	Endemic to China; Zhejiang, Guizhou, and Yunnan	VU
*Cipangopaludina longispira*	Heude (1890)	Lake snail	Endemic to China; N, SE, and SW	VU
*Cipangopaludina ussuriensis*	Gerstfeld (1859)	River snail	Major range in China; NE	VU
*Cipangopaludina ventricosa*	Heude (1890)	Lake snail	Endemic to China; Guizhou and Yunnan	VU
*Cipangopaludina yunnanensis*	Zhang et al. (1981)	Pond snail	Endemic to China; Yunnan	VU
*Cipangopaludina menglaensis*	Zhang et al. (1981)	River snail	Endemic to China; Yunnan	EN
*Cipangopaludina dianchiensis*	Zhang (1994)	Lake snail	Endemic to China; Yunnan	CR
*Cipangopaludina hainanensis*	Kebelt (1909)	River snail	Endemic to China; Yunnan	EX
*Cipangopaludina leucostoma*	Heude (1890)	Pond snail	Endemic to China; Guangxi and Yunnan	EN
*Filopaludina bengalensis*	Lamarck (1822)	Lake snail and river snail	Minor range in China; Hainan and Yunan	EN
*Simopaludina munensis*	Brandt (1974)	River snail	Minor range in China; Yunan	CR
*Bellamya quadrata*	Benson (1982)	Lake snail and pond snail	Major range in China; Yunnan, N, SE	LC
*Bellamya angularis*	Muller (1773)	Lake snail	Endemic to China; N, SE, and SW	LC
*Bellamya purificata*	Heude (1890)	Lake snail and pond snail	Endemic to China; widely distributed in China	LC
*Bellamya aeruginosa*	Reeve (1863)	Lake snail and pond snail	Endemic to China; widely distributed in China except NE	LC
*Bellamya limnophila*	Mabille (1886)	Lake snail	Endemic to China; Hubei and Yunnan	EN
*Bellamya dispiralis*	Heude (1890)	Lake snail and river snail	Endemic to China; N, SE, and SW	LC
*Bellamya boettger*	Heude (1890)	Lake snail	Endemic to China; Jiangxi, Hunan, and Guangdong	EN
*Bellamya delavayana*	Heude (1890)	Lake snail	Endemic to China; Sichuan and Yunnan	EN
*Bellamya lithophaga*	Heude (1890)	River snail	Endemic to China; Zhejiang and Anhui	EN
*Bellamya lapillorm*	Heude (1890)	Lake snail and river snail	Endemic to China; SE, Sichuan	VU
*Bellamya manhungensis*	Zhang et al. (1994)	Pond snail	Endemic to China; Yunnan	EN
*Bellamya papillapicala*	Zhang et al. (1982)	Lake snail	Endemic to China; Hunan	CR
*Bellamya heudei*	Dautzenberg and Fisher (1905)	Lake snail and river snail	Endemic to China; Anhui, S	VU
*Bellamya heudei*	Dautzenberg and Fisher (1905)	Lake snail and river snail	Endemic to China; Jiangxi, Guangdong, and Hainan	VU
*Bellamya lapidea*	Heude (1890)	Lake snail and river snail	Endemic to China; SE, N, Sichuan	LC
*Bellamya fantozatiana*	Heude (1890)	Lake snail	Endemic to China; Hubei	EX
*Bellamya smith*	Yen (1942)	River snail	Endemic to China; S	EN
*Bellamya turritus*	Yen (1939)	River snail	Endemic to China; Fujian, Guangdong, and Yunan	EN
*Bellamya demolite*	Heude (1890)	River snail	Endemic to China; Guangdong and Anhui	EN
*Mekangia hunanensis*	Yen (1942)	Lake snail and river snail	Endemic to China; Hunan	EX
*Mekangia rivularia*	Keblet (1909)	Lake snail River snail	Endemic to China; S, Shandong	LC
*Angulyagra polyzonata*	Franeufeld (1862)	Lake snail and pond snail	Endemic to China; S, Yunnan	LC
*Angulyagra oxytropis*	Heude (1890)	Lake snail, river snail, and pond snail	Endemic to China; Yunnan	VU
*Angulyagra mutica*	Keblf (1909)	Pond snail	Endemic to China; Guangdong, Guangxi, and Yunnan	EN
*Angulyagra thersites*	Reeve (1863)	Pond snail	Endemic to China; Guangdong	EX
*Angulyagra annulatus*	Yen (1939)	River snail	Endemic to China; Hunan and Guizhou	EX
*Angulyagra subcostata*	Gray (1939)	Pond snail	Endemic to China; Guangdong and Hainan	EN
*Angulyagra costata*	Quoy and Gaimard (1832)	Pond snail	Minor range in China; Guangdong	CR
*Margarya melanoides*	Nevil (1877)	Lake snail	Endemic to China; Yunnan	VU
*Margarya yaugtsunghaiensis*	Tchang and Tsi (1949)	Lake snail	Endemic to China; Yunnan	CR
*Margarya monodi*	Dautzenberg and Fisher (1903)	Lake snail	Endemic to China; Yunnan	EN
*Angulyagra elongata*	Tchang and Tsi (1949)	Lake snail	Endemic to China; Yunnan	EX
*Angulyagra Yini*	Tchang and Tsi (1949)	Lake snail	Endemic to China; Yunnan	EX
*Angulyagra mansuyi*	Dautzenberg and Fisher (1906)	Lake snail	Endemic to China; Yunnan	VU
*Angulyagra bicostata*	Tchang and Tsi (1949)	Lake snail	Endemic to China; Yunnan	EN
*Angulyagra tchangsii*	Xia (1982)	Lake snail	Endemic to China; Yunnan	EX
*Angulyagra franchetti*	Mabille (1886)	Lake snail	Endemic to China; Yunnan	EX
*Angulyagra tropidophora*	Mabille (1886)	Lake snail	Endemic to China; Yunnan	EN
*Angulyagra carinata*	Neumayr (1883)	Lake snail	Endemic to China; Yunnan	EN
*Rivularia auriculata*	Martans (1875)	River snail	Endemic to China; Hunan, Jiangxi, and Guangdong	LC
*Rivularia elongata*	Heude (1890)	River snail	Endemic to China; SE	LC
*Rivularia globosa*	Heude (1890)	River snail	Endemic to China; SE, Sichuan	VU
*Rivularia ovum*	Heude (1890)	River snail	Endemic to China; SE, Sichuan	LC
*Rivularia glandina*	Heude (1890)	River snail	Endemic to China; Jiangxi and Hunan	EN
*Rivularia vucarubata*	Keblt (1909)	River snail	Endemic to China; Hunan	EN
*Rivularia calcarata*	Keblt (1909)	River snail	Endemic to China; Hunan	EX
*Rivularia porcellanca*	Keblt (1909)	River snail	Endemic to China; Hunan and Hubei	EX
*Rivularia subelliptica*	Heude (1890)	River snail	Endemic to China; Anhui	EX

Note: Ex, extinct; EW, extinct in the wild; RE, regionally extinct; CR critically endangered; EN, endangered; VU, vulnerable; NT, near threatened; LC, least concern; DD, data deficient; NA, not applicable

Recently, there have been several novel studies on *C. chinensis* and *C. cathayensis* snails. Zhou et al. (2022a) first utilized single-molecule real-time (SMRT) sequencing technology to conduct a full-length transcriptome analysis of *C. chinensis*. They analyzed enriched metabolic, signal transduction, and immune-related pathways, identifying several candidate genes related to drug metabolism and immune response. In the same year, Zhao et al. (2022b) found a significant amount of aryl sulfatase and β-glucuronidase in *C. chinensis*. These enzymes are crucial for the cleavage process of bound natural estrogens (C-NEs). Liu et al. (2023) compared the shell protein characteristics of *P. canaliculata* (*P. canaliculata*) and *C. chinensis* in China. They discovered that carbonic anhydrase was absent in the shells of both snails, suggesting that freshwater snails may have a unique method of regulating the calcification process, which differs significantly from the shell mineralization of marine mollusks.There are also two studies on *C. cathayensis*. Jiang et al. (2022) found that exposure to cadmium (Cd) can disrupt the balance of intestinal flora in *C. cathayensis* in natural rivers in Guangxi Province. This can alter cellular metabolic processes, affecting the community structure, function of intestinal flora, and intestinal homeostasis Using high-throughput sequencing, Wu et al. (2022) revealed that high-temperature treatment can alter the intestinal flora characteristics in *C. cathayensis* and inhibit some carbohydrate metabolic pathways, which may be involved in disease-associated pathways such as systemic lupus erythematosus, *Vibrio cholerae*, hypertrophic cardiomyopathy, and shigellosis . Consequently, high temperatures affect the community structure and function of *C. cathayensis’s* intestinal flora, providing a new approach to studying its intestinal flora’s response mechanism to global warming.

Although there have been several reports on transcriptome analysis, shell protein characteristics, and intestinal flora in Viviparidae snails, little research has been conducted in proteomics and phosphoproteomics. In this study, we aimed to analyze and compare the proteomics profiles of *C. chinensis* carcass from different geographical areas and environmental conditions. As one of the relatively new proteomic methods, we employed this approach to analyze the differential proteomic profiles of *C. chinensis*. Furthermore, our results may provide novel insights into proteins and signaling pathways in Viviparidae. The comprehensive research will offer invaluable information regarding metabolic pathways and biological functions and potentially reveal novel food nutrition and medicinal components in *C. chinensis*.

## 2 Materials and methods

### 2.1 Samples

**FIGURE 1:**
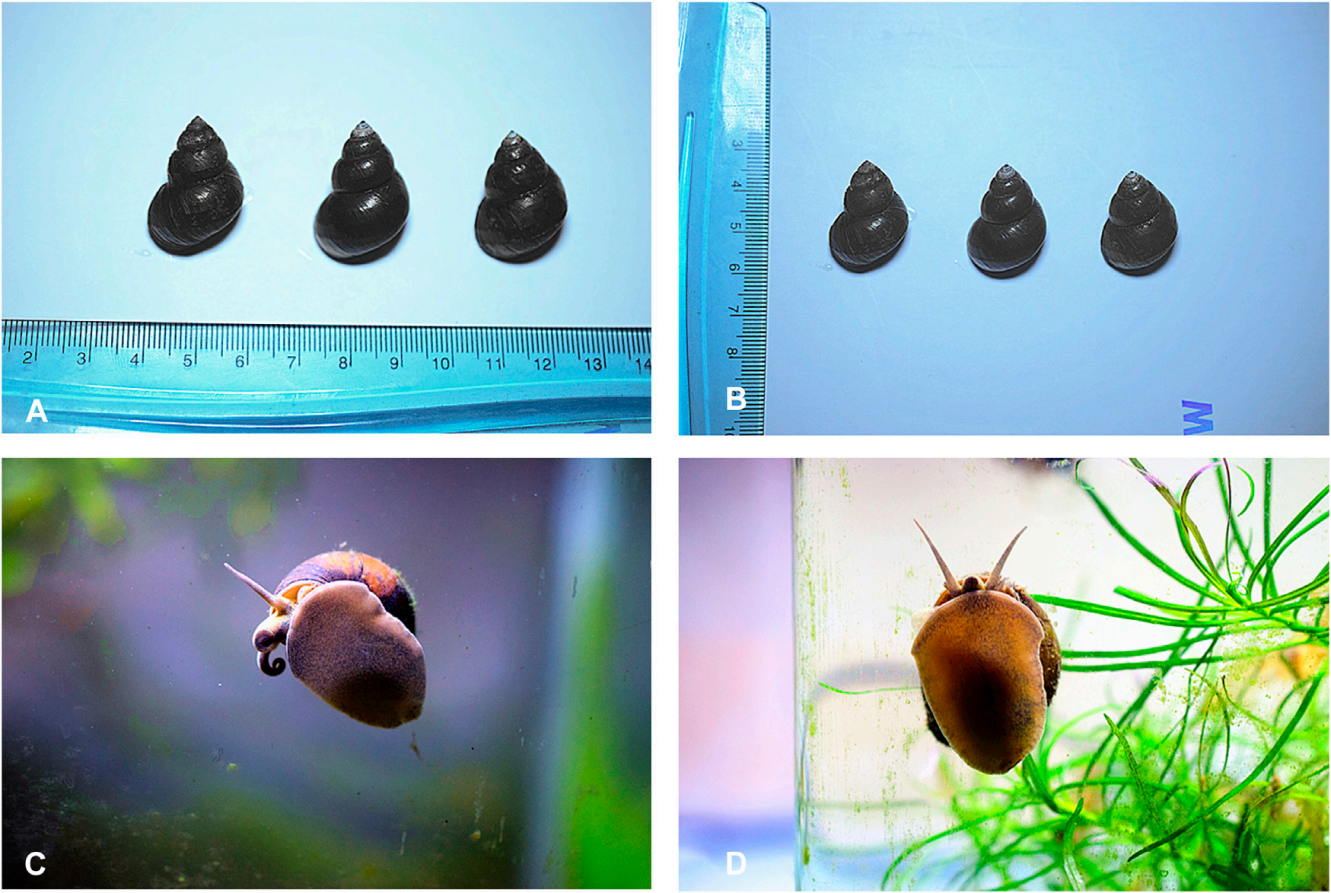
Female and male *C. chinensis*. **(A, B)** The size of partial snail samples (2.8 cm × 1.5 cm). **(C)** The two antennae of the male snail are asymmetrical, and the right antennae are short and thick, which is the specialized mating organ. **(D)** The antennae of the female snail are symmetrically homomorphic.

The groups were divided into A, B, and C, with each group originating from a different geographical area in Shandong Province, China. Each group comprised three female *C. chinensis* from the same area, and we selected one representative snail for proteomic analysis. Single female *C. chinensis* (2.8 cm × 1.5 cm) from each group were collected and pooled to eliminate the effects of individual differences (Figure 1). The female snails were collected from three locations along the Yi River in Shandong Province, China. These locations exhibited apparent environmental differences in soil characteristics and conditions (north latitude 34°22′∼36°13′, east longitude 117°24′∼119°11′, belonging to a temperate monsoon climate). We used *C. chinensis* carcasses (muscle) as protein samples for proteomic analysis. All protein samples were taken from the same part of the carcass. All samples were immediately transported on dry ice.

### 2.2 High-performance liquid chromatography fractionation

The samples were concentrated, digested, and extracted, and the protein concentration was determined by the Bradford protein assay. The processing of protein samples was referenced from the previous methods (Liu et al., 2022). The supernatant from each sample, containing precisely 0.1 mg of protein, was digested using Trypsin Gold (Promega) at a 1:50 enzyme-to-substrate ratio. After 16 h of digestion at 37°C, a portion of all peptide samples was mixed equally to form the mixture sample. Then, all samples were desalted using a C18 cartridge to remove urea, and the desalted samples were dried via vacuum centrifugation. The mixed sample was fractionated using a C18 column (Waters BEH C18, 4.6 mm × 250 mm, 5 µm) on a Shimadzu LC 15 HPLC operating at 0.6 mL/min. Mobile phases A (2% acetonitrile, adjusted pH to 10.0 using ammonium hydroxide) and B (98% acetonitrile, adjusted pH to 10.0 using ammonium hydroxide) were used to develop a gradient elution. The eluates were monitored at UV 214 nm, collected into separate tubes every minute, and finally merged. All fractions were dried under vacuum centrifugation and reconstituted in 0.1% (v/v) formic acid (FA) in water.

### 2.3 Liquid chromatography–tandem mass spectrometry analysis

For transition library construction, shotgun proteomic analyses were performed using an UltiMate 3000 Ultra High-Performance Liquid Chromatography (UHPLC) System (Dionex) coupled with a Q Exactive HF Mass Spectrometer (Thermo Fisher). Twenty components were dissolved in 20 µL of nano-LC A solution. A 2 µL of the sample was injected into the column gradient and eluted, analyzed, and identified using an Orbitrap Elite Mass Spectrometer. The sample was analyzed using a Thermo Nano LC 1000 high-performance liquid chromatography (HPLC) system, with an Acclaim PepMap^®^ 100 C18, 3 μm, 100 Å (75 μm × 2 cm) sample column and an Acclaim PepMap^®^ RSLC C18, 2 μm, 100 Å (50 μm × 15 cm). The detailed procedures were referenced from previous methods (Liu et al., 2022).

### 2.4 Phosphorylation analysis

For the enrichment of phosphopeptides, an appropriate amount of PuriMag Si–TiO_2_ Magnetic Beads was transferred into a 1.5 mL tube, and the sample buffer (1 M glycolic acid in 80% acrylonitrile (ACN) and 5% trifluoroacetic acid (TFA)) was washed three times. The sample buffer was added, mixed thoroughly, and incubated at room temperature for 20 min. After incubation, the supernatant was then transferred to a new tube. The supernatant was washed once with sample buffer, twice with washing buffer (80% ACN and 1% TFA), and again (10% ACN and 0.2% TFA) washed two times with washing buffer. Finally, the phosphopeptide was eluted with 5% NH_4_OH, and the eluent was acidified using formic acid. After secondary enrichment, immobilized metal affinity chromatography (IMAC) enrichment was carried out.

A measure of 200 μL of IMAC beads was moved into a 1.5 mL tube and centrifuged at 2,100 *g* for 30 s, and then the stored buffer was removed. Microbeads were washed with 6% acetic acid for 30 s; 0.1 M of FeCl_3_ with 6% acetic acid was added to the tube and incubated for 2 h, and then beads were washed with 6% acetic acid three times. The mixed buffer (250 mM acetic acid and 30% ACN) was formulated into a 50% suspension for the next use. The dried peptide was re-suspended in the slurry at a concentration of 1 μg/μL and incubated at room temperature with vigorous shaking for 1 h. After incubation, the supernatant was transferred to a new tube. The beads were washed with buffer solution twice and then washed again with water once. Then, the phosphopeptide was eluted with 5% of NH_4_OH, and the eluent was acidified using formic acid. After the second enrichment of the incubation solution, the sample was desalted. Finally, the eluent enriched by PuriMag Si–TiO_2_ and IMAC was mixed, and desalted vacuum freeze-drying was performed after completion.

### 2.5 Bioinformatic analysis

The identified proteins were classified by Gene Ontology (GO) annotation. The GO annotation proteome was derived from the UniProt–GOA database (http://www.ebi. ac.uk/GOA/). The Kyoto Encyclopedia of Genes and Genomes (KEGG) database was used to annotate protein pathways. According to the online KEGG Automatic Annotation Server (KAAS), the protein’s KEGG database was annotated and mapped using the KEGG pathway database.

## 3 Results

### 3.1 LC–MS/MS identification proteins

**FIGURE 2:**
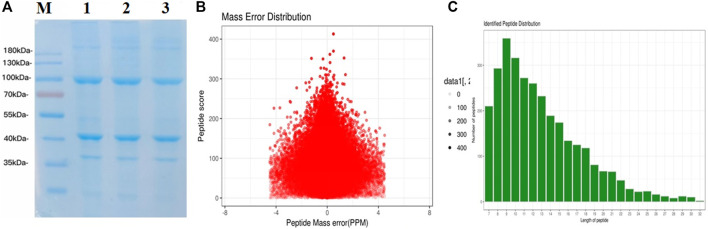
Total identified peptide distribution. **(A)** SDS-PAGE of groups A, B, and C. **(B)** Mass error distribution (PPM). **(C)** Identified peptide distribution.

A total of 1,382 *C. chinensis* proteins were identified, with 690 proteins quantified. Among these, 172 (168), 196 (174), and 159 (154) differentially expressed proteins (DEPs) were significantly upregulated (downregulated) among pairwise comparisons (A vs B, A vs C, and B vs C) (Figure 1; Figure 2).

### 3.2 Functional classification of differentially expressed proteins

**FIGURE 3:**
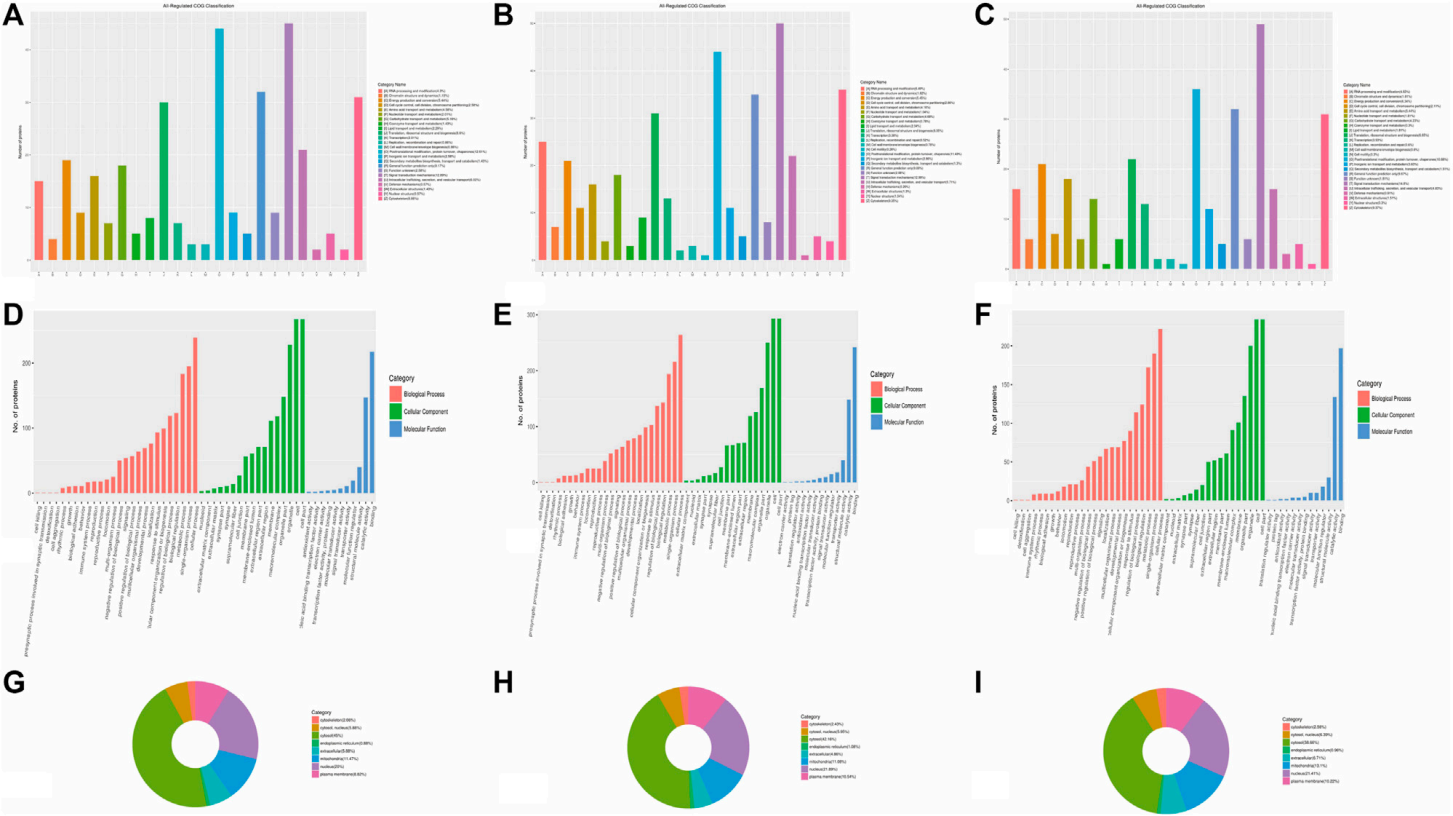
Categories of DEPs involved in cellular components, molecular functions, biological processes, and subcellular locations. **(A–C)** COG classification of all regulated DEPs in A vs B, A vs C, and B vs C, respectively. **(D–F)** Categories of DEPs in cellular components, molecular functions, and biological processes in A vs B, A vs C, and B vs C, respectively. **(G–I)**: Subcellular locations of DEPs in A vs B, A vs C, and B vs C, respectively.

DEPs were defined based on the threshold protein quantification (fold change ≥1.2 and *p*-value ≤0.05). Functional classification results indicated that all regulated DEPs were associated with cellular components, molecular functions, biological processes, and subcellular locations. A total of 340 DEPs from the A vs B group were primarily categorized as signal transduction mechanisms (12.89%), post-translational modification, protein turnover, and chaperones (12.61%), and cytoskeletons (8.85%) (Figure 3A). Additionally, 370 DEPs from the A vs C group showed similar trends to the A vs B group (Figure 3B). Finally, 313 DEPs from the B vs C group were primarily categorized as signal transduction mechanisms (14.8%), post-translational modification, protein turnover, and chaperones (10.88%), general function only (9.67%), and cytoskeletons (9.37%) (Figure 3C). This distribution differs from the A vs B and A vs C groups.

DEPs primarily participate in cellular components such as cells, cell parts, organelles, organelle parts, macromolecular complexes, membranes, membrane-enclosed lumens, membrane parts, extracellular regions, extracellular region parts, cell junctions, supramolecular fibers, extracellular matrices, extracellular matrix components, synapse parts, synapses, and nucleoids (the green column in Figures 3D–F). These DEPs also play significant roles in binding, catalytic activity, and structural molecular activity in terms of molecular function (the blue column in Figures 3D–F). Furthermore, over 10 types of DEPs are involved in biological processes, including cellular processes, single-organism processes, metabolic processes, biological regulation, regulation of biological processes, cellular component organization or biogenesis, response to stimulus, localization, developmental processes, and multicellular organismal processes (the orange column in Figures 3D–F). The subcellular locations of all the regulated proteins mainly concentrate in the cytosol (ranging from 38.66% to 45%), nucleus (ranging from 20% to 21.89%), mitochondria (ranging from 11.08% to 13.10%), and plasma membrane (ranging from 8.85% to 10.54%). Each component percentage exhibits minimal differences among the three groups (Figures 3G–I).

**FIGURE 4:**
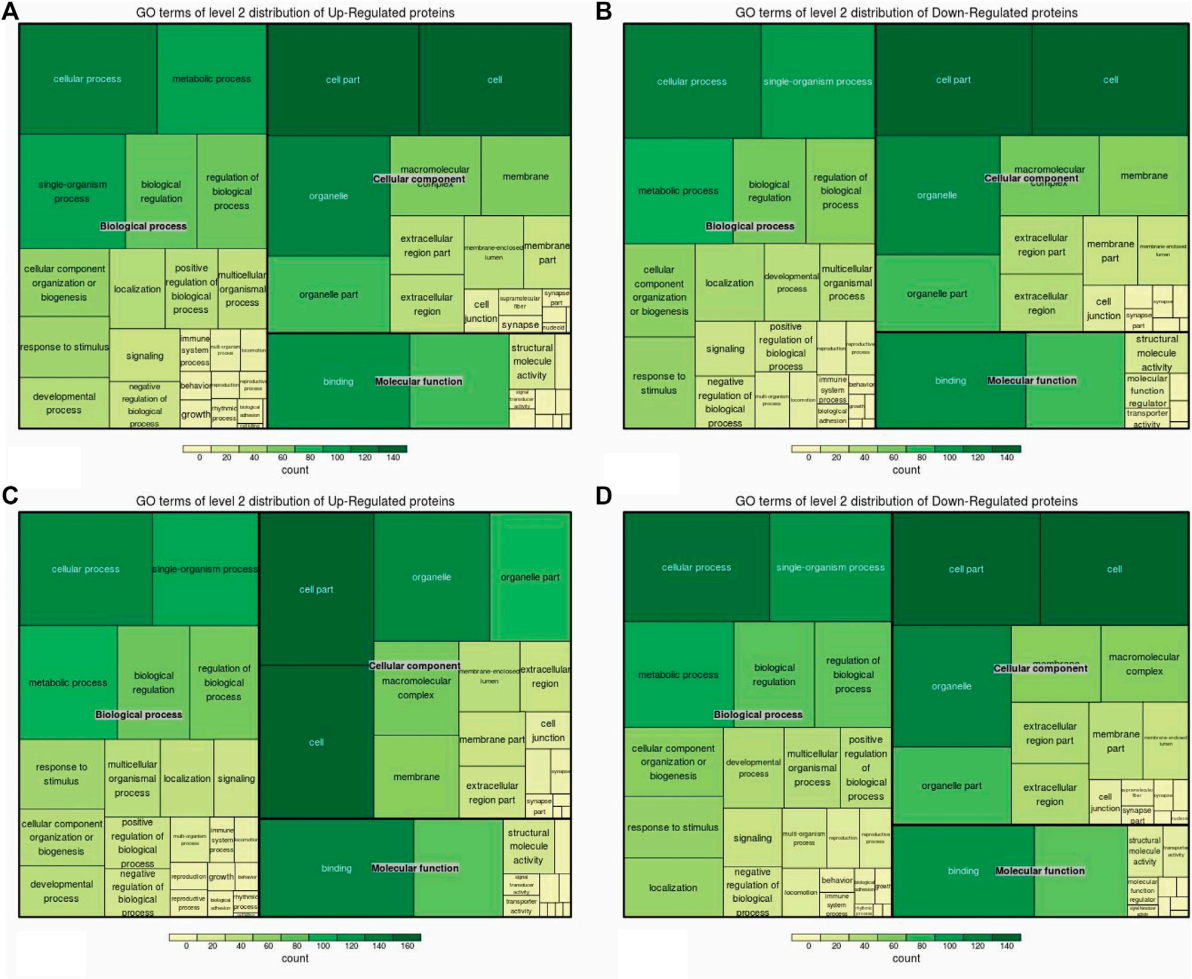
Up/downregulated DEPs involved in functional classification. **(A, C)** Upregulated proteins involved in the classification of A vs B and A vs C, respectively. **(B, D)** Downregulated proteins involved in the classification of A vs B and A -vs- C, respectively.

The number of upregulated and downregulated proteins involved in signal transduction mechanisms exceeds 20 in all three groups (A vs B, A vs C, and B vs C), particularly in the A vs C comparison, where the number exceeds 30 (Figures 4C,D). Additionally, the number of downregulated proteins involved in posttranslational modification, protein turnover, and chaperones also exceeds 20 (Figure 4B). However, only the number of upregulated proteins in the A vs B comparison exceeds 20 (Figure 4A). Notably, over 20 cytoskeleton proteins are involved in the upregulated processes in the A vs C comparison (Figure 4C).

### 3.3 Functional enrichment of DEPs

**FIGURE 5:**
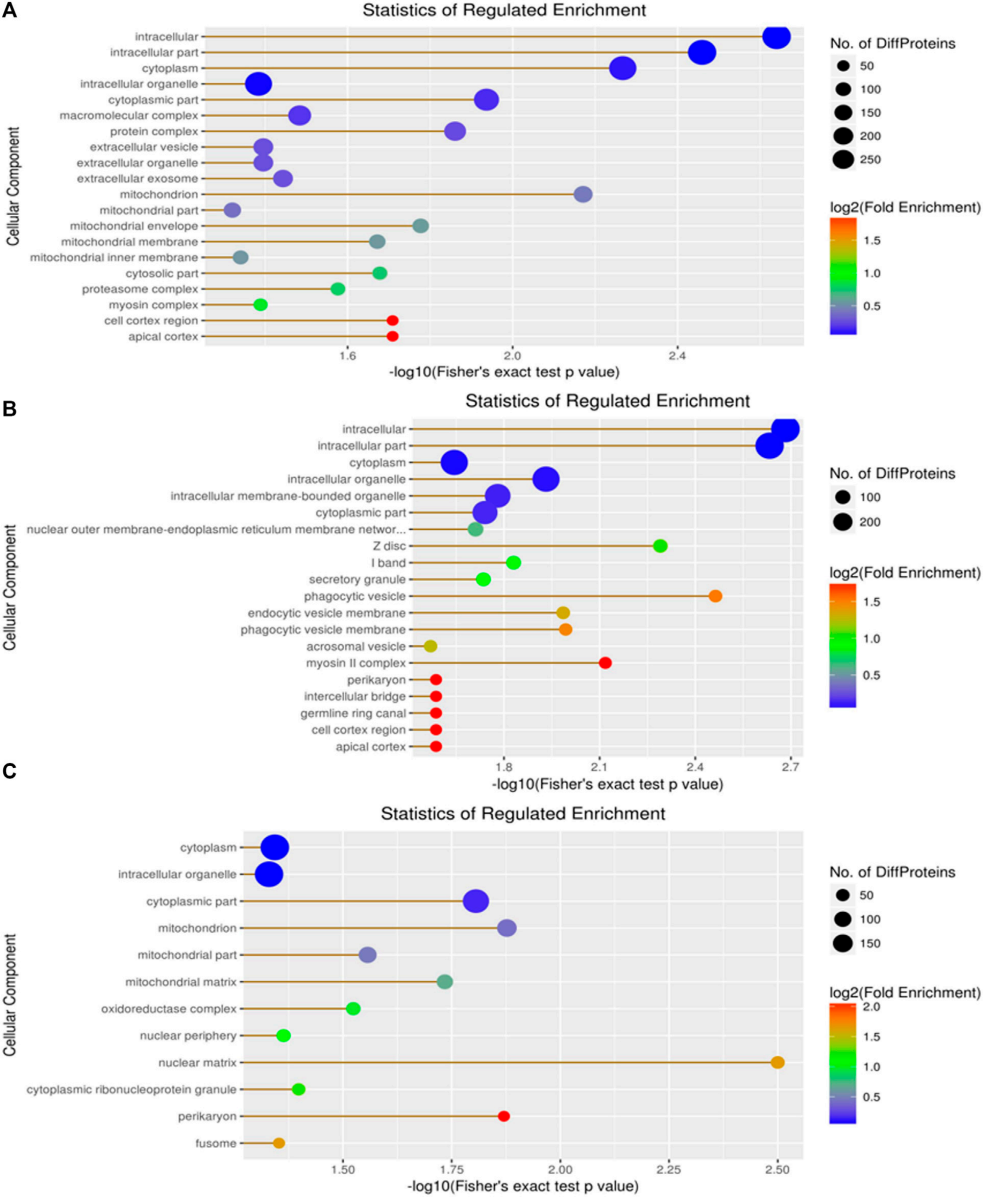
Visualization of significantly regulated GO cellular component enriched maps. **(A)** GO cellular component map for all regulated DEPs in A vs B; **(B)** GO cellular component map for all regulated DEPs in A vs C; **(C)** GO cellular component map for all regulated DEPs in the B vs C group.

Cellular components, molecular functions, biological processes, and the KEGG were used for the enrichment analysis of the DEPs. Cellular component enrichment results revealed 276 DEPs among 1,022 proteins in the A vs. B group, with 137 upregulated and 139 downregulated DEPs (Figure 5A). Initially, we calculated the enrichment of all regulated GO cellular components for the DEPs in the A vs B group. The proteins localized to the intracellular, intracellular part, cytoplasm, and intracellular organelle regions exceeded 250 (Figure 5A). Compared to the A vs B group, cellular component enrichment analysis in the A vs C group revealed 303 DEPs among 1,022 proteins, including 160 upregulated and 143 downregulated DEPs, respectively (Figure 5B). The number of proteins localized to the intracellular, intracellular part, cytoplasm, intracellular organelle, and cytoplasm regions exceeded 200 (Figure 5B). Finally, cellular component enrichment analysis in the B vs C group revealed 244 DEPs among 1,022 proteins, with 123 upregulated and 121 downregulated DEPs (Figure 5C). The number of proteins localized to the cytoplasm, intracellular organelle, and cytoplasm regions, as well as each type of DEP, exceeded 150 (Figure 5C).

**FIGURE 6:**
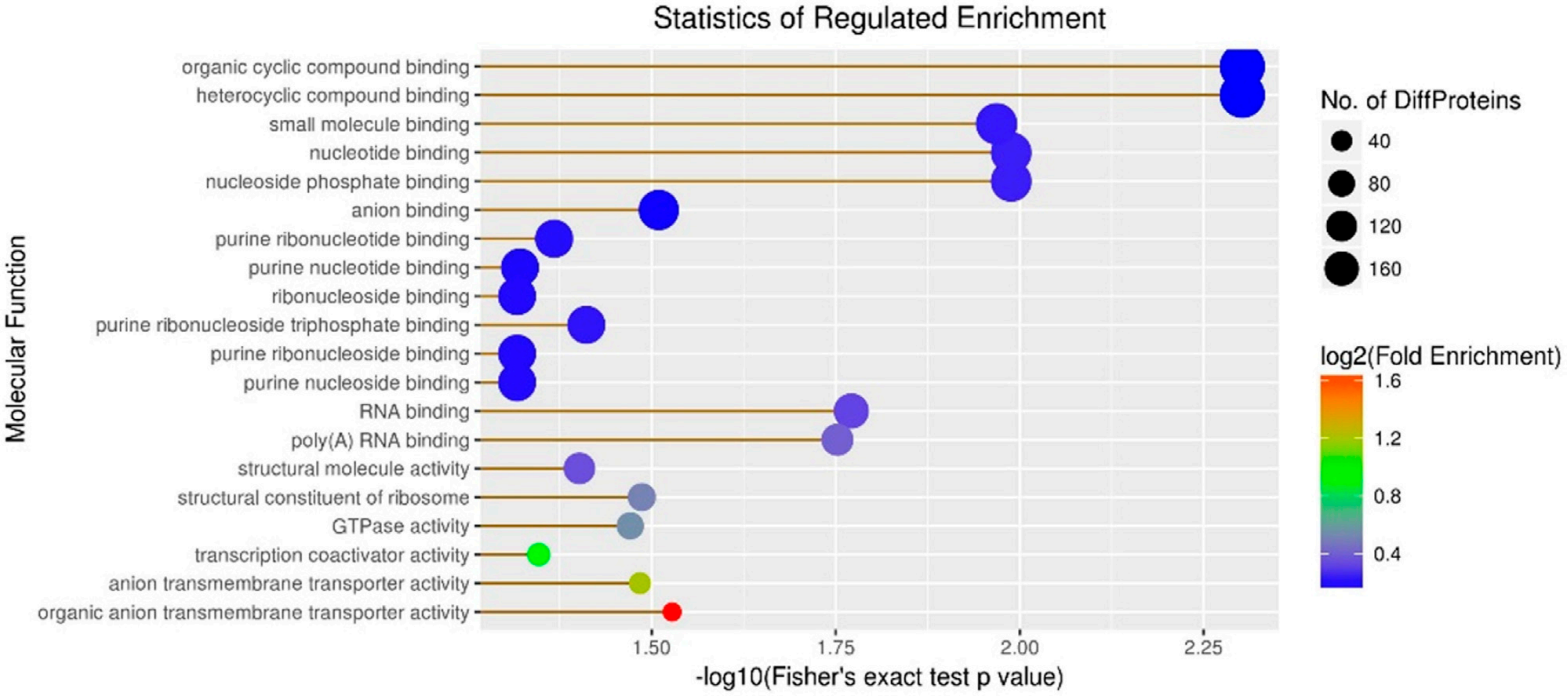
Visualization of all regulated GO molecular function enrichment maps in the A vs C group.

Subsequently, molecular function enrichment results revealed 278 DEPs among 983 proteins in the A vs B group, comprising 135 upregulated and 143 downregulated DEPs (Figure 6). Initially, we calculated the GO molecular function enrichment of all regulated DEPs in the A vs B group. The top two enriched functions were small-molecule binding and anion binding, with over 100 proteins in each category (Figure 6). Then, our analysis of the A vs C group revealed 305 DEPs among 983 proteins, including 167 upregulated and 138 downregulated DEPs (Figure 6). Over 12 enriched functions were identified, including organic cyclic compound binding, heterocyclic compound binding, small molecule binding, nucleotide binding, nucleoside phosphate binding, anion binding, purine ribonucleotide binding, purine nucleotide binding, ribonucleoside binding, purine ribonucleoside triphosphate binding, purine ribonucleoside binding, and purine nucleoside binding, with all these functions represented by over 160 proteins (Figure 6). Finally, the B vs C group showed 247 DEPs among 983 proteins, including 130 upregulated and 117 downregulated DEPs (Figure 6). The top two enriched functions were ion binding and small molecule binding, with the number of proteins in these three categories exceeding 100 (Figure 6).

**FIGURE 7:**
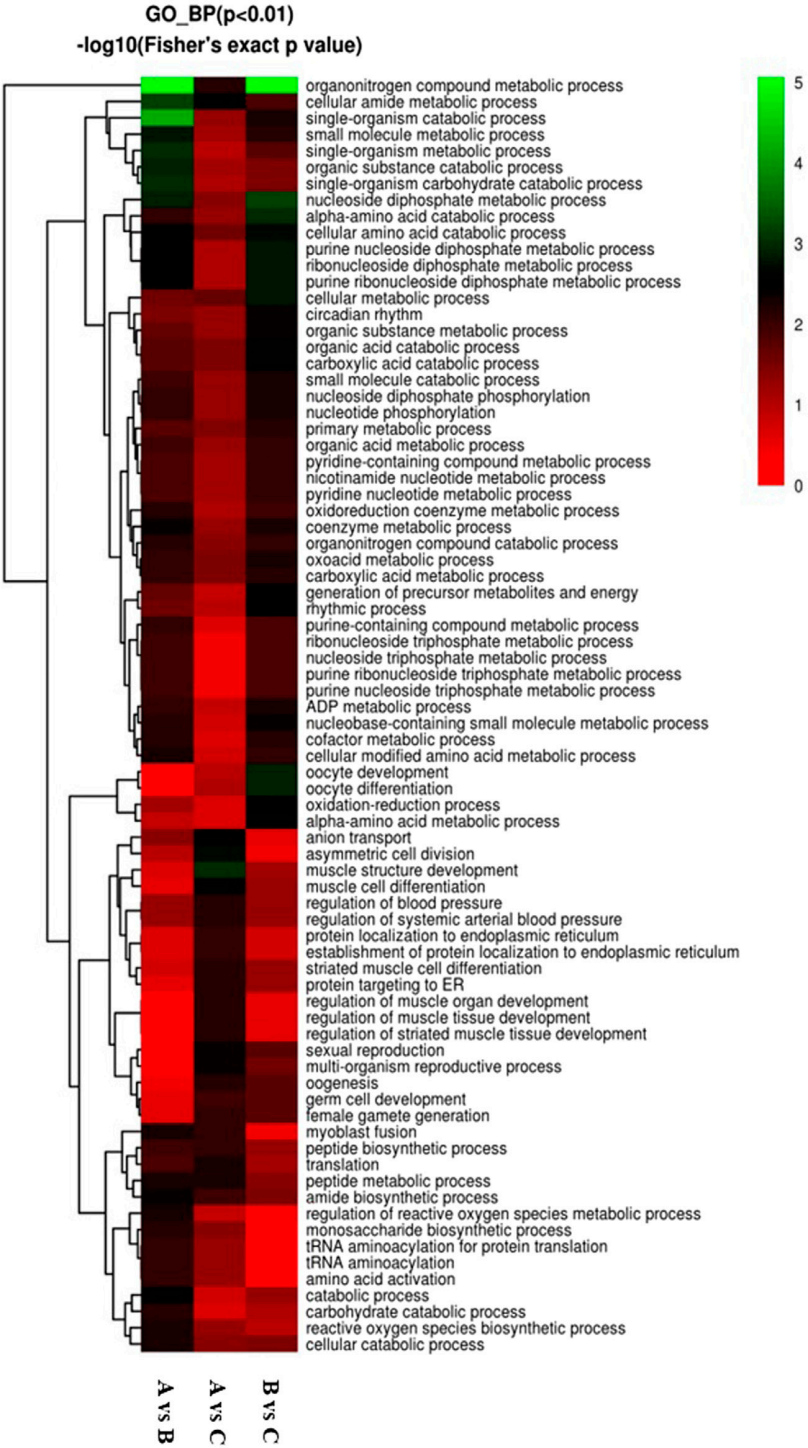
Visualization of significant biological processes enriched maps. **(A)** Biological processes map for all regulated DEPs in A vs B; **(B)** Biological processes map for all regulated DEPs in A vs C; **(C)** Biological processes map for all regulated DEPs in the B vs C group..

GO enrichment analysis was also conducted for DEPs associated with biological processes in three groups. Our results revealed that 258 DEPs out of 968 proteins, comprising 129 upregulated and 129 downregulated DEPs, were identified in the A vs B group (Figure 7A). These DEPs were primarily involved in single-organism metabolic, organo-nitrogen compound metabolic, small-molecule metabolic, catabolic, organic substance catabolic, and cellular catabolic processes (Figure 7A). In comparison to the A vs B group, GO enrichment analysis of DEPs showed 282 DEPs out of 968 proteins in the A -vs- C group, with 146 upregulated and 136 downregulated DEPs (Figure 7B). The top five DEPs were mainly involved in single-organism metabolic, oxoacid metabolic, organic acid metabolic, and carboxylic acid metabolic processes (Figure 7B). Finally, GO enrichment analysis of DEPs revealed 239 DEPs out of 968 proteins in the B vs C group, with 116 upregulated and 123 downregulated DEPs (Figure 7C). The top two DEPs were associated with oxoacid metabolic and single-organism catabolic processes, with both types of DEPs numbering more than 25 (Figure 7C).

**FIGURE 8:**
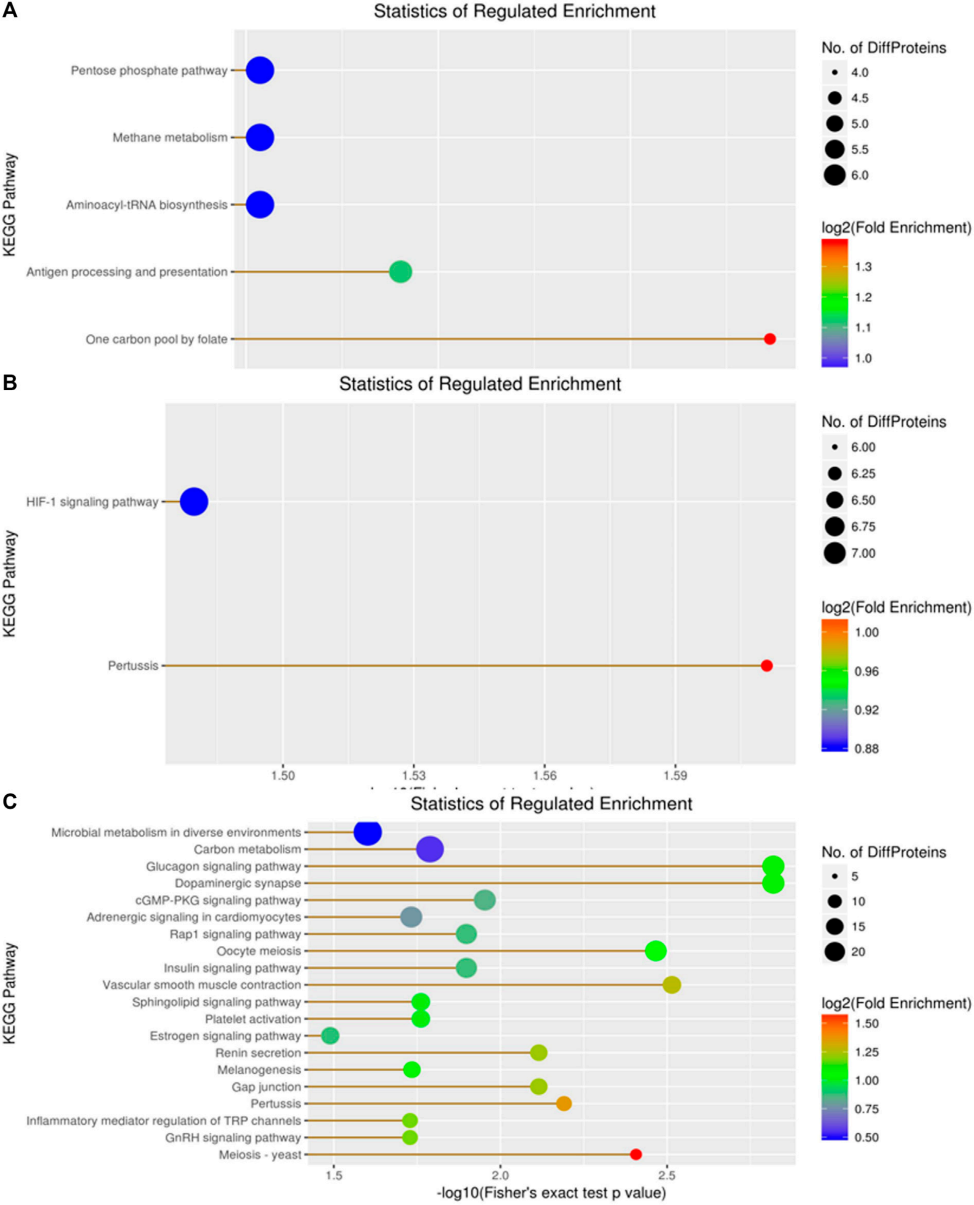
Visualization of significantly regulated KEGG Pathways enriched maps. **(A)** KEGG Pathways map for all regulated DEPs in A vs B; **(B)** KEGG Pathways map for all regulated DEPs in A vs C; **(C)** KEGG Pathways map for all regulated DEPs in the B vs C group..

KEGG enrichment analysis revealed 159 DEPs among 414 proteins, with 66 upregulated and 93 downregulated DEPs in the A vs B group (Figure 8A). The top three DEPs were involved in the pentose phosphate pathway, methane metabolism, and aminoacyl-tRNA biosynthesis in diverse environments (Figure 8A). In contrast, the KEGG enrichment analysis of the A vs C group revealed 175 DEPs among 414 proteins, comprising 89 upregulated DEPs and 86 downregulated DEPs (Figure 8B). Among these, only one DEP was involved in the HIF-1 signaling pathway. For the B vs C group, the KEGG enrichment analysis revealed 138 DEPs among 414 proteins, with 77 upregulated and 61 downregulated DEPs (Figure 8B). Compared to the previous two groups, the top two DEPs in this group were mainly involved in metabolic pathways and microbial metabolism in diverse environments (Figure 8C).

**FIGURE 9:**
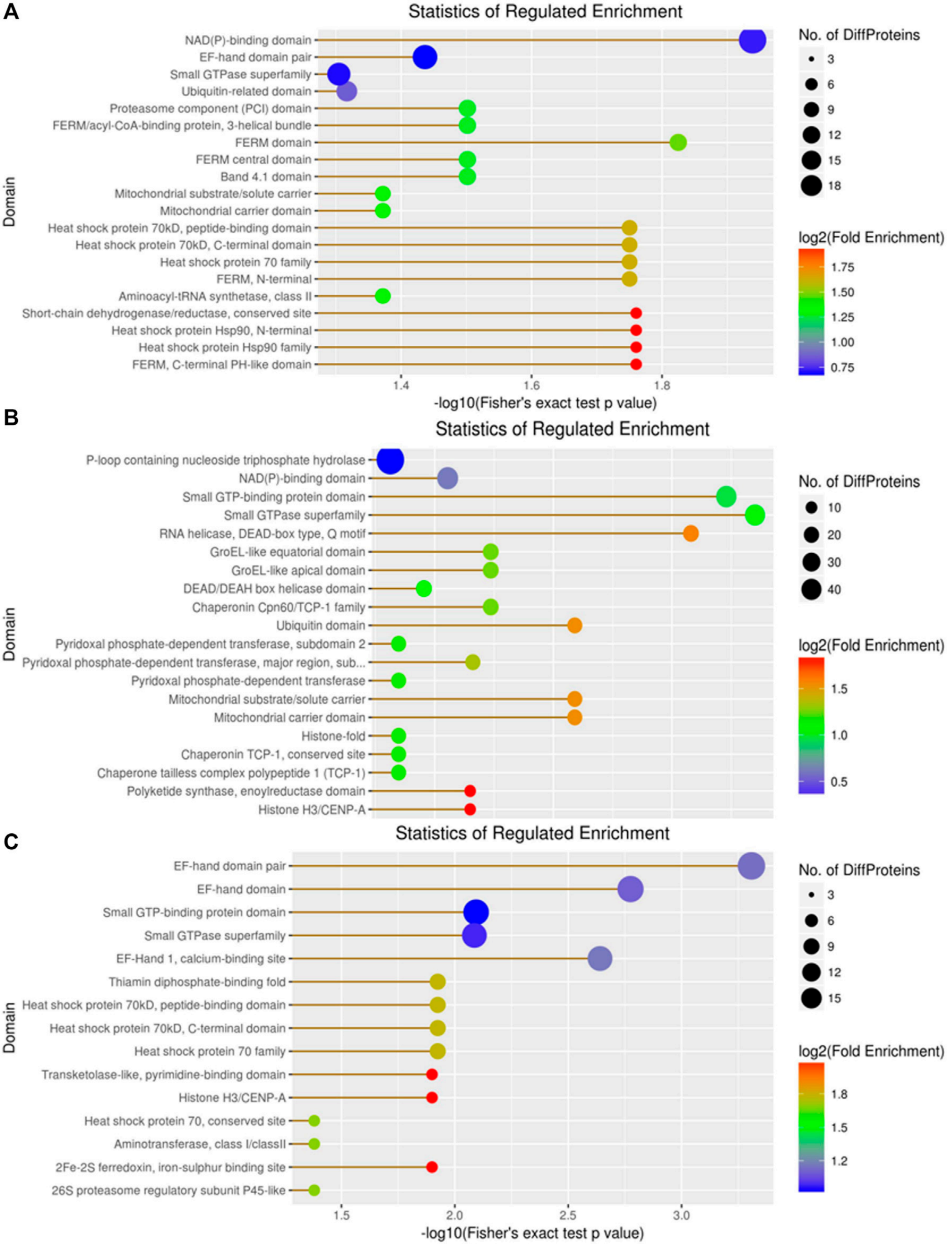
Visualization of significant DEP domains in the **(A)** vs **(B)**, **(A)** vs **(C)**, and **(B)** vs **(C)** groups.

In the domain enrichment analysis, we first identified 327 DEPs among 1,260 quantitatively analyzed proteins, comprising 164 upregulated and 163 downregulated DEPs. The results revealed several important functional domains, including the NAD(P)-binding domain, EF-hand domain pair, small GTP-binding protein domain, and ubiquitin-related domain, in the A vs B group (Figure 9). Next, we analyzed the domain enrichment in the A vs C group, where 356 DEPs were identified among 1,260 quantitatively analyzed proteins, with 189 upregulated and 167 downregulated DEPs. The results showed that the number of P-loop containing nucleoside triphosphate hydrolase and NAD(P)-binding domains was higher than that of other domains (Figure 9). Finally, in the B vs C group, we identified 294 DEPs among 1,260 quantitatively analyzed proteins, with 151 upregulated and 143 downregulated DEPs. This group displayed several important functional domains, including the EF-hand domain pair, EF-hand domain, and small GTP-binding protein domain, which are similar to those found in the A vs B group (Figure 9).

### 3.4 Phosphoproteomic analysis

A total of 1,039 phosphorylated proteins were identified and quantified in *C. chinensis*, with 118 (77), 125 (92), and 101 (103) significantly upregulated (downregulated) DEPs detected among the pairwise comparison groups. There were 2,914 phosphorylated modification sites, primarily including three key sites: serine, threonine, and tyrosine. Notably, several novel phosphorylated proteins and uncharacterized proteins were involved in regulating signaling pathways in *C. chinensis*. KEGG enrichment analysis revealed several novel signaling pathways, such as the Rap1 signaling pathway, Ras signaling pathway, calcium signaling pathway, and longevity-regulating pathway (see Supplementary Tables S1–3).

## 4 Discussion

In our research, several novel proteins were discovered in *C. chinensis* from China. Caenogastropoda is the largest extant group of gastropod mollusks, comprising 136 families, most of which are various sea and freshwater snails (Colgan et al., 2007). Viviparidae (Gray, 1847) is the most widely distributed freshwater family of Neogastropoda, distributed globally except for Antarctica and South America, including 150 species from 31 genera (Stelbrink et al., 2020). According to traditional taxonomy, there are approximately 70 species of Chinese Viviparidae, which are classified into 9 genera: Viviparus, Filopaludina, Mekongia, Angulyagra, Rivularia, Siamopaludina, Margarya, Bellamya, and Cipangopaludina. Cipangopaludina and Bellamya are widely distributed in rivers and lakes in China (Liu, 1979; Liu, 1991). *Cipangopaludina yunnanensis* (*C. yunnanensis*), *Cipangopaludina menglaensis* (*C. menglaensis*), and C*ipangopaludina dianchiensis* (*C. dianchiensis*) (Table 1) are the most unique endemic species found in Yunnan Province, China (Zhang, 1949; Zhang, 1990; Du, 2013).

There are numerous species of Viviparidae snails reported in China, including *C. chinensis*, *C. cathayensis*, *Cipangopaludina ventricosa* (*C. ventricosa*), *C. ussuriensis*, *Bellamya purificata* (*B. purificata*), *Bellamya aeruginosa* (*B. aeruginosa*), *Bellamya quadrata* (*B. quadrata*), *Margarya melanioides* (*M. melanioides*), *Margarya yangtsunghaiensis* (*M. yangtsunghaiensis*), and *Rivularia auriculata* (*R. auriculata*) (Table 1). Due to the environmental damage caused by rapid industrial development and urbanization, the species distribution and population size of mollusks in China have been severely threatened (Wang et al., 2011), leading to many indigenous mollusk species becoming endangered or even extinct (Shu et al., 2010). To protect and restore these indigenous snail species, scholars worldwide have conducted in-depth studies on the classification, phylogeny, life history, and population genetics of snails.

Viviparidae are the primary group of freshwater macrobenthos in lakes and rivers. In particular, the species of Cipangopaludina and Bellamya are widely distributed and serve as crucial indicators of water quality. Studies have reported that Viviparidae can significantly remove ammonium salts, nitrates, and nitrites from water through bioturbation and physiological activities like absorption, transformation, degradation, and excretion of nutrient salts (Zhang and Zhejiang, 2011; Chen, 2012). Through secretions, the suspended particles in the water rapidly flocculate into clumps, accelerating the sedimentation of suspended substances and reducing their content in the water (Zhou, 2012). Therefore, the Viviparidae family plays an indispensable role in maintaining the ecological health of water bodies and restoring eutrophic water bodies.

With the advancement in molecular biology research, especially the application of genomics and transcriptomics in studying the adaptive evolution and genetic variation of mollusks, it has become increasingly evident that their robust habitat adaptation is closely tied to genetic evolution mechanisms. Related studies have encompassed heat stress (Wang et al., 2014), immunity (Shiel et al., 2015), salinity adaptation (Meng et al., 2013), neuroregulation (Moroz et al., 2006), pesticide metabolism (Mu et al., 2015), and genetic differentiation (Sun et al., 2015). Nonetheless, reports on the proteomics of freshwater gastropod mollusks remain scarce (Mu et al., 2017; Escobar-Correas et al., 2023).

Based on the proteomic analysis of *C. chinensis*, our research identified a total of 1,382 proteins, with 690 proteins being quantified. Among these, 172 (168) and 196 (174) proteins were significantly upregulated (downregulated) in different geographical isolates, respectively. Additionally, 159 (154) proteins exhibited significant regulation in another comparison. Concurrently, we also identified and quantified 1,039 phosphorylated proteins in *C. chinensis*. Among these, 96 (78), 104 (79), and 90 (91) proteins were significantly upregulated (downregulated) in three distinct groups. Notably, several novel and uncharacterized proteins were found to be involved in the upregulated or downregulated phosphorylated signaling pathways in *C. chinensis* (Supplementary Tables S1–3).

**FIGURE 10:**
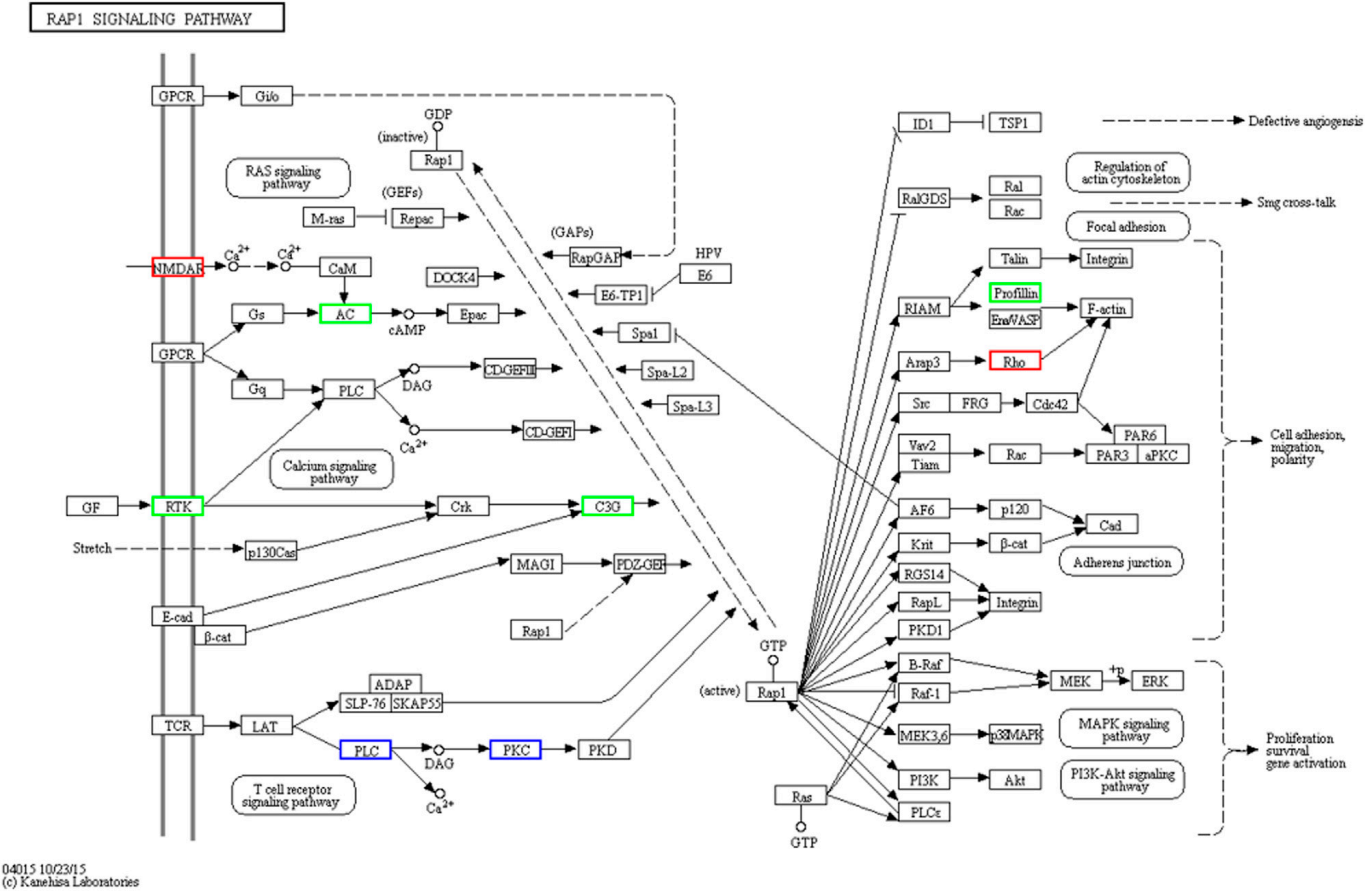
Visualization of the RAP1 signaling pathway. Rap1 is a small GTPase that controls diverse processes, such as cell adhesion, cell–cell junction formation, and cell polarity. Like all G proteins, Rap1 cycles between an inactive GDP-bound and an active GTP-bound conformation. A variety of extracellular signals control this cycle through the regulation of several unique guanine nucleotide exchange factors (GEFs) and GTPase activating proteins (GAPs).

Our results indicate that guanylate cyclase (Pirahanchi and Dimri, 2023), a protein with diverse functions, may be involved in heart failure (Kansakar et al., 2021; Liang and Liang, 2023), gastrointestinal disorders (Waldman and Camilleri, 2018; Makrynitsa et al., 2019), visceral pain (Hannig et al., 2014), colorectal cancer (Aka et al., 2017), nutraceuticals (McCarty et al., 2022), and Ca (2+) signal transduction (Duda et al., 2014). However, there is little research focused on its role in Viviparidae snails. The KEGG enrichment analysis revealed that guanylate cyclase is potentially involved in the Rap1 signaling pathway (Figure 10), Ras signaling pathway, calcium signaling pathway, longevity-regulating pathway, glutamatergic synapse, Circadian entrainment, and gap junction. Notably, several species, including humans, mice, rats, bovines, and zebrafish, have been implicated in the Rap1 signaling pathway. Rap1 plays a pivotal role in regulating cell–cell and cell–matrix interactions by modulating the function of integrins and other adhesion molecules in various cell types. Furthermore, Rap1 regulates MAP kinase (MAPK) activity in a cell-type-dependent manner. Proteins such as profilin (Bailly, 2004), receptor protein tyrosine kinase (Qi et al., 2019), Ras-GEF domain-containing protein (Smit et al., 1996; Mun and Jeon, 2012), and guanylate cyclase domain-containing protein are all components of the Rap1 signaling pathway. qRT-PCR results have demonstrated that miR-31-5p target genes in lung squamous cell carcinoma (LUSC) are significantly associated with signal transduction, transmembrane receptor protein tyrosine-kinase activity, and the Rap1 signaling pathway (Chi et al., 2019). Studies on differentially expressed aberrantly methylated hub genes in breast cancer have shown that downregulated and hypermethylated genes are involved in transmembrane receptor protein tyrosine-kinase activity and the Rap1 signaling pathway (Qi et al., 2019). Recent research has found that Rap1 controls actin cytoskeletal reorganization and interacts with RacGEF1 through the Rac signaling pathway (Smit et al., 1996; Mun and Jeon, 2012).

Although we use proteomics methods to select several novel DEPs in *C. chinensis*, however, there are still some limitations in the study. Firstly, we didn't send large number of samples for testing, few samples were collected in our research because of lacking adequate funding. Secondly, the samples only from Spring but not represent all seasons in one year, which the up-regulated or down-regulated proteins in proteomics profile may have changes in different seasons. Thirdly, more proteins samples detection could take represent the complete proteomics and phosphoproteomics data in *C. chinensis*. Although our study has successfully conducted the proteomics and phosphoproteomics in *C. chinensis*, there still need to be more research grant to study the molecular function, biological progresses, and signaling pathway in the future. We plan to carry out more research on biological characteristics in *C. chinensis.*

Although we employed proteomic methods to identify several novel DEPs in *C. chinensis*, there are still some limitations to our study. First, we did not submit a large number of samples for testing due to a lack of adequate funding, which resulted in a relatively small sample size. Second, the samples were collected only in the spring and do not represent all seasons in a year. Therefore, the upregulated or downregulated proteins identified in our proteomic profile may vary in different seasons. Third, detecting more protein samples could provide a more comprehensive representation of proteomic and phosphoproteomic data in *C. chinensis*. Although we have successfully conducted proteomic and phosphoproteomic analyses in *C. chinensis*, further research is needed to investigate the molecular functions, biological processes, and signaling pathways in more depth. We plan to carry out additional research to investigate the biological characteristics of *C. chinensis*.

## 5 Conclusion

The present study provides insight into the proteomic and phosphoproteomic profiles of *C. chinensis* carcasses. Although we just adopted the spring season to analyze the identified proteins, samples from different seasons may show different expressed protein changes. According to our research, each single *C. chinensis* displays differences in different areas and environments. GO and KEGG analyses showed that DEPs play an important role in metabolic processes, localization, and biological regulation. The phosphorylated proteins may be involved in signaling pathways and protease regulation, such as guanylate cyclase, tyrosine protein kinase, receptor protein tyrosine kinase, and glyoxylate reductase/hydroxypyruvate reductase. Therefore, our research will help improve our understanding of food nutrition, signaling pathways, and metabolic mechanisms in *C. chinensis*.

## Data Availability

The datasets presented in this study can be found in online repositories. The names of the repository/repositories and accession number(s) can be found at: https://wwwiprox.cn, with the dataset identifier PXD046762.
