# Supplementary material for: Proteomics and phosphoproteomics reveal novel proteins involved in Cipangopaludina chinensis carcasses
Source: Front Chem. 2024 Aug 29;12:1416942. doi: 10.3389/fchem.2024.1416942 (PMC11390518; doi:10.3389/fchem.2024.1416942)
Supplement: Supplementary file 1 [file Table1.DOCX]

Supplementary Table1. All regulated DEPs involved in signaling pathway in *Cipangopaludina chinensis* (A-vs-B).

| Category_  Name | Description OS=  *Cipangopaludina chinensis* | ProteinIDs | Regulated-Stage |
| --- | --- | --- | --- |
| Rap1 signaling pathway | Profilin;  Receptor protein-tyrosine kinase;  Ras-GEF domain-containing protein;  Guanylate cyclase domain-containing protein | A0A2T7PRC6;  A0A2T7PNG5;  A0A2T7NMD8;  A0A2T7PNF2 | Down |
| Insulin secretion | Guanylate cyclase domain-containing protein;  G-protein coupled receptors family 1 profile domain-containing protein;  Gastrin/cholecystokinin type B receptor | A0A2T7PNF2;  A0A2T7NNZ7;  A0A2T7PP57 | Down |
| Shigellosis | Profilin;  Tyrosine-protein kinase | A0A2T7PRC6;  A0A2T7NM72 | Down |
| Calcium signaling pathway | Guanylate cyclase domain-containing protein;  G-protein coupled receptors family 1 profile domain-containing protein;  Gastrin/cholecystokinin type B receptor | A0A2T7PNF2;  A0A2T7NNZ7;  A0A2T7PP57 | Down |
| cAMP signaling pathway | Receptor ligand binding region domain-containing protein;  Uncharacterized protein;  Sodium/potassium-transporting ATPase subunit alpha | A0A2T7Q0R0;  A0A2T7NSU1;  A0A2T7P2D7 | Up |
