# Supplementary figures and images for: Proteomics and phosphoproteomics reveal novel proteins involved in Cipangopaludina chinensis carcasses
Source: Front Chem. 2024 Aug 29;12:1416942. doi: 10.3389/fchem.2024.1416942 (PMC11390518; doi:10.3389/fchem.2024.1416942)

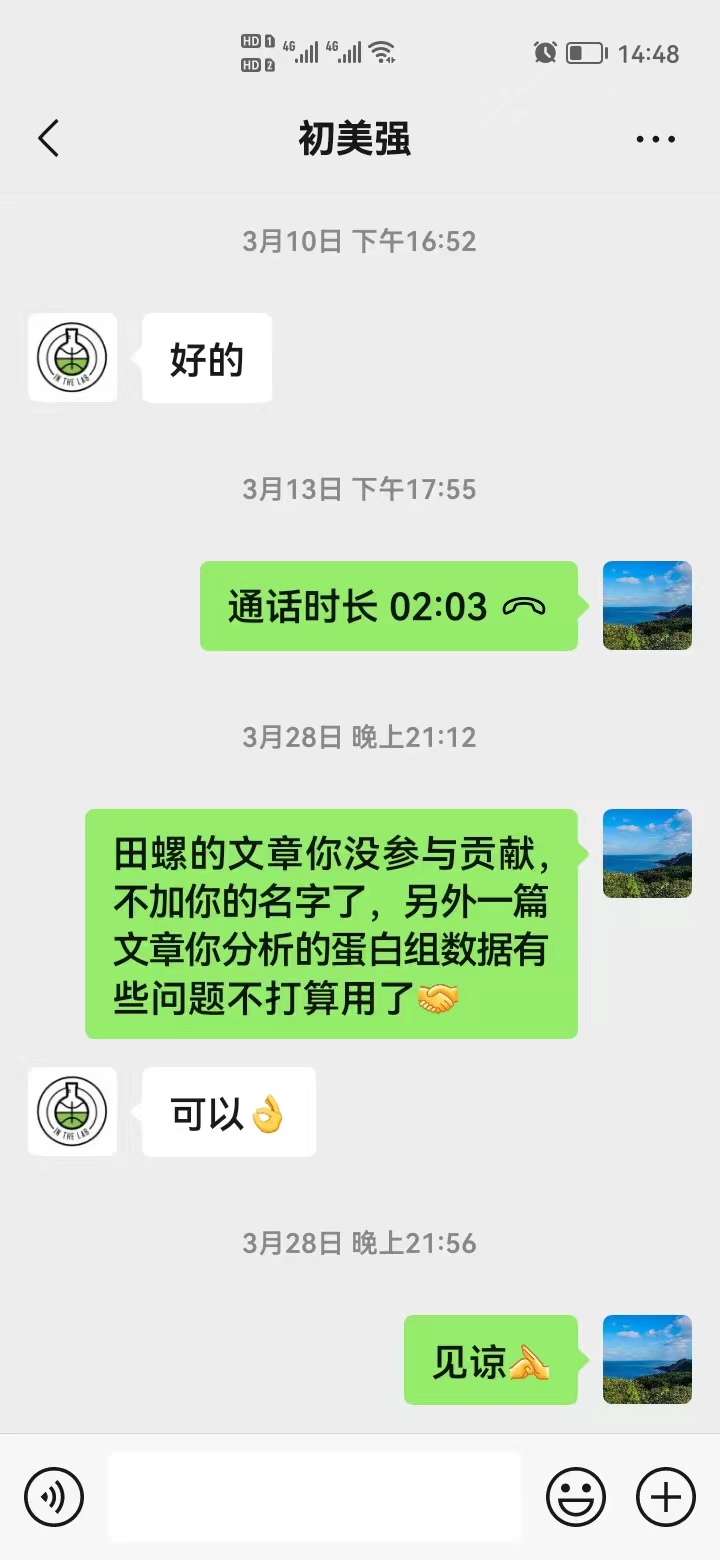

Supplement: Supplementary file 2 [file Image1.JPEG]
